# Nonsurgical Treatment of Class III Malocclusion with Both Anterior and Posterior Crossbites Combined with Impacted and Congenitally Missed Teeth

**DOI:** 10.1155/2020/8825212

**Published:** 2020-09-07

**Authors:** Yahya A. Alogaibi, Ahmed R. Afify, Ahmad A. Al-Fraidi, Ali A. Hassan

**Affiliations:** ^1^Bisha Dental Center, Ministry of Health, P.O. Box 418, Bisha 61922, Saudi Arabia; ^2^Department of Orthodontic, King Fahad Hospital, Specialized Dental Center, Madina, Saudi Arabia; ^3^Orthodontic Department, Faculty of Dentistry, King Abdulaziz University, P.O. Box 80209, Jeddah 21589, Saudi Arabia; ^4^Orthodontic Department, Faculty of Dentistry, Mansoura University, Mansoura, Egypt; ^5^Alfarabi Private College, Jeddah, Western Region, Saudi Arabia; ^6^Department of Orthodontics, Faculty of Dentistry, King Abdulaziz University, P.O. Box 80209, Jeddah 21589, Saudi Arabia

## Abstract

Class III malocclusions present a great challenge for many orthodontists, especially if malocclusions are found in adult patients and alongside other dental problems. This case report shows an adult patient with a skeletal class III anterior crossbite, a unilateral posterior crossbite on the right side, a congenital absence of both lateral incisors and retained deciduous teeth, and shift in the lower midline. The upper retained deciduous teeth and lower premolars were extracted. Leveling and alignment were initiated. Build-up composite resin placed on the first molars allowed for bite opening. The crossbites were corrected by using sequentially larger archwires combined with class III elastics until both the anterior and posterior crossbites were corrected. The impacted upper right canine was exposed using the closed eruption technique and leveled into the position of the upper lateral incisor. Miniscrews were utilized to close the residual spaces. Both canines were reshaped to simulate the upper lateral incisors. At the end of the treatment, good esthetic and functional results were obtained. In conclusion, orthodontic camouflage can be a viable option for treating patients with multiple skeletal and dental problems.

## 1. Introduction

Treatment of class III malocclusions in adult patients can be a great challenge, especially in borderline cases where both camouflage and orthognathic surgeries are possible lines of treatment [1, 2]. The outcome of treatment in these cases will depend on proper diagnosis of the problem, i.e., whether it is skeletal or dental and the severity of the problem [3]. Recently, many orthodontic treatment mechanics were able to produce orthognathic-like results in adult class III malocclusions by utilizing Temporary Anchorage Devices (TADs) [4, 5]. The use of TADs could avoid the need for orthognathic surgery, especially when the patient refuses such treatment [6].

Crossbites can be classified, according to their positon, into anterior or posterior crossbites [7]. Multiple anterior crossbites may suggest anteroposterior maxillary deficiency and/or mandibular excess [8]; on the other hand, posterior crossbite can reflect transverse maxillary deficiency [9]. It is uncommon to see a combination of these two types; however, if this occurs, this would strongly suggest an overall deficiency of the maxilla and/or overgrowth of the mandible [9, 10].

Upper canine impaction is one of the most commonly seen problems in orthodontics [11]. The method of treatment usually depends on the position, depth, and angulation of impaction [11]. The etiology of this impaction can be explained by two main theories: the genetic theory and the guidance theory. The genetic theory suggests that impaction of upper canines occurs due to the expression of multiple genes that lead to congenital anomalies and the absence of an upper lateral incisor [12, 13]. On the other hand, the guidance theory, as its name implies, states that canine impaction occurs due to an absence of guidance during eruption, which is gained from the root of the lateral incisor [14].

In this case report, we describe the nonsurgical treatment of an adult patient suffering from a skeletal class III malocclusion combined with anterior and posterior unilateral crossbites, an impacted upper right canine, and a congenitally missing upper right lateral incisor.

## 2. Diagnosis

**Figure 1:**
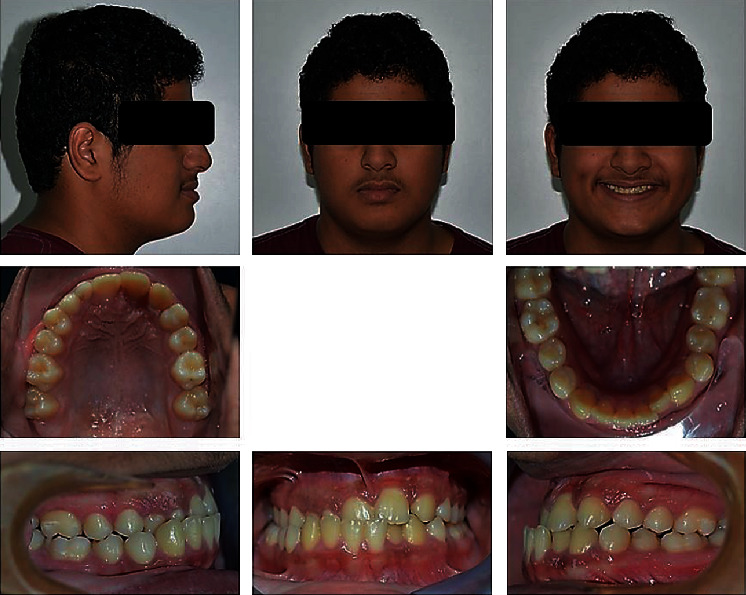
Pretreatment extraoral and intraoral photographs of the patient.

**Figure 2:**
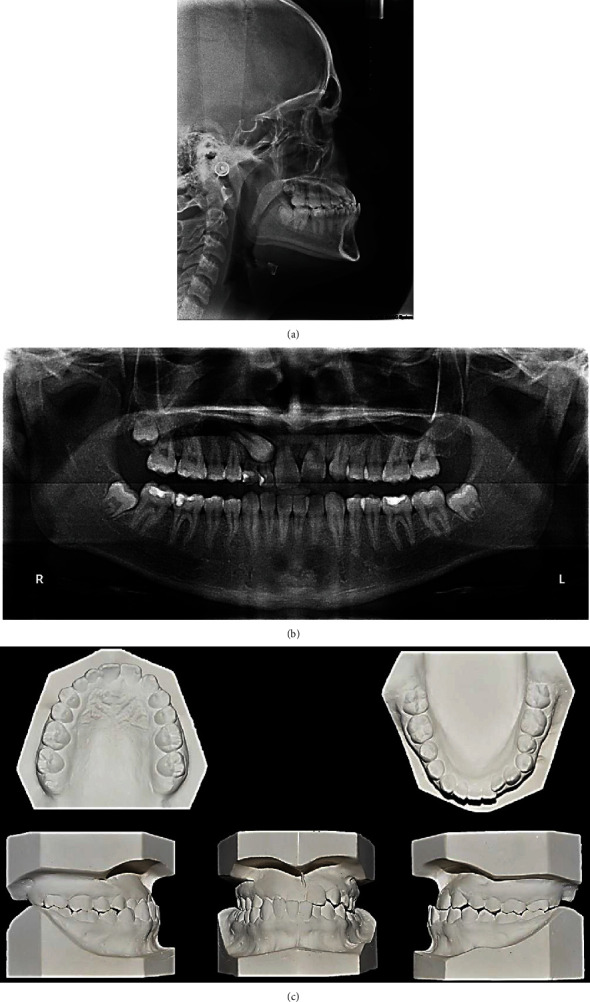
Pretreatment cephalometric radiograph, panoramic radiographs, and study models: (a) cephalometric radiograph, (b) panoramic radiographs, and (c) study models.

A 17-year-old male presented to the orthodontic clinic, and his chief complaint was “I want to fix my crooked teeth.” Intraoral examination revealed fair oral hygiene, plaque accumulation, and staining around his teeth. The patient had a mild class III skeletal base with a class II canine in the right side, a congenital absence of both lateral incisors, retained upper right deciduous lateral incisor, and a canine with an impacted upper right permanent canine. This was complicated with functional shift, also anterior and unilateral posterior crossbites on the right side. Additionally, the patient had 2 mm spacing in the maxillary arch and 2 mm crowding in the mandibular arch with a lower midline that was shifted to the right by 2 mm. Moreover, cephalometric analysis (Table 1) showed a class III skeletal base, normal vertical skeletal relationship, proclined upper incisors, normal inclination of the lower incisors, and final stage of growth maturation (cervical vertebral maturation stage 5), indicating an absence of any remaining growth. A panoramic radiograph showed an impacted upper right permanent canine and a congenitally missing upper right lateral incisor and upper left third molar (Figures 1 and 2(a)–2(c)). Secondary caries were detected on LL6, LR6, and LR7.

## 3. Treatment Objectives

The proposed treatment objectives were as follows: [1] reinforcing oral hygiene and caries control, [2] bringing the impacted canine into the line of occlusion, [3] correcting both anterior and posterior crossbites, and [4] correcting the lower midline shift and alleviation of mandibular arch crowding.

## 4. Treatment Plan

Two options of treatment were available:

1. First option is an orthognathic surgery to correct the transverse and the anteroposterior skeletal discrepancies
2. Second option is an orthodontic treatment alone through the following procedure: regarding the mandibular arch, extraction of lower first premolars and space closure

In the maxillary arch, two options were available: the first option was to substitute the congenitally missing lateral incisor with the impacted canine and to advance the buccal segment to close the space of the canine on the right side. The second option was to guide the canine into its normal position on the right side in addition to opening a space between the upper left canine and central incisor to place an implant or bridge to restore the upper laterals.

After discussing the advantages and disadvantages of each option with the patient and considering the priorities of esthetic and functional demands, orthodontic treatment alone with the substitution of the congenitally missing laterals with canines was approved and other significant procedures mentioned in the (Table 2) were taken into consideration as well.

## 5. Progress of Treatment

The treatment was initiated by extraction of the retained upper right deciduous lateral, canine, and lower premolars. Treatment was initiated by banding the first molars and bonding of the other teeth using 0.018 slot preadjusted edgewise brackets with Roth prescription. Build-up composite resin was applied on both of the lower first molars. Leveling and alignment were done by 0.014^″^ Niti wire followed by 0.016^″^ Niti, then 0.016 × 0.022 Niti^″^, a 0.016 × 0.016^″^ stainless steel wire, and finally a 0.016 × 0.022^″^ stainless steel wire.

The extraction spaces in the mandibular arch were initially closed by utilizing class III elastics. The maxillary arch was expanded gradually using sequentially larger archwires until the function shift and both the anterior and posterior crossbites were corrected. After the initial alignment of the maxillary arch, the impacted upper right canine was exposed surgically and a gold chain was attached to its labial surface; afterward, a closed eruption technique was utilized.

**Figure 3:**
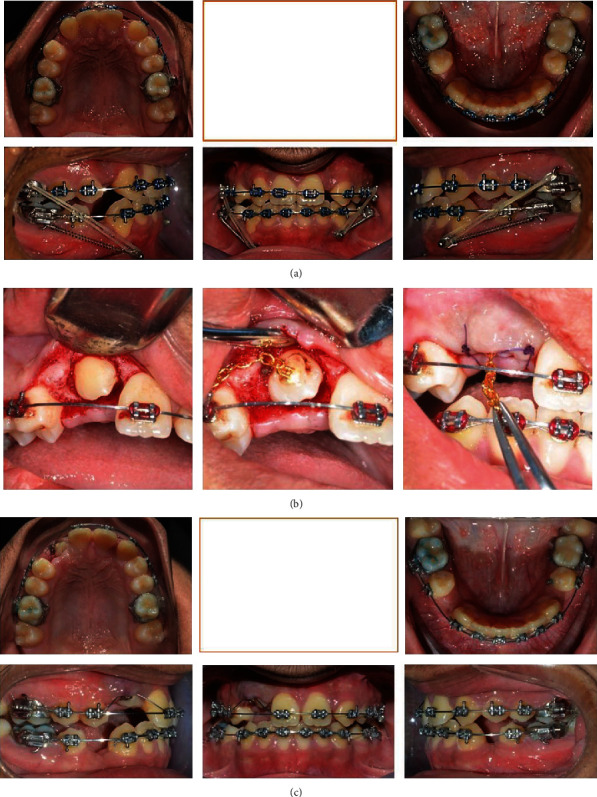
Multiple progress photographs (a–c).

As the canine came near to the maxillary arch, a “piggy-back technique” was utilized by the insertion of an auxiliary wire of 0.012^″^ Niti combined with a 0.016 × 0.016^″^ stainless steel wire (Figure 3). To close the spaces in the maxillary and mandibular arches, two miniscrews (1.6 mm diameter and 8 mm length, RMO ®, Denver, USA) were inserted between the lower canines and laterals on both sides. Elastics were first utilized to close the remaining spaces between the lower second premolars and the canine by attaching it from the lower first molar to the miniscrews on each side. This was followed by attaching the elastics to the upper molars to advance the buccal segment and close the anterior spaces in the maxillary arch. Finally, an upper closing T-loop (0.016 × 0.022^″^ stainless steel wire) was used to close the remaining space.

The maxillary arch was further expanded posteriorly by expanding the stainless steel wires. During finishing, a labial root torque was placed on the first premolars to mimic the canines and palatal root torque for correct crown positioning and to reduce buccal prominence of the canine root in order to mimic laterals. In contrast to the first premolars, a palatal root torque was placed on the canines to mimic the laterals. Selective grinding was done for both of the canines to remove their prominent cusp tips and reshape them as lateral incisors. Finally, box elastics were used posteriorly for interdigitation. The total treatment duration was 26 months. Retention was accomplished by a wraparound retainer for the maxillary arch and a Hawley retainer for the mandibular arch.

## 6. Treatment Results

**Figure 4:**
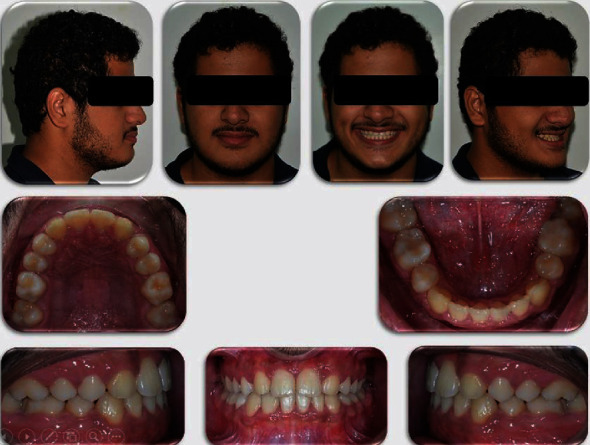
Posttreatment photographs of the patient.

The treatment resulted in improved facial esthetics and masticatory functions. A class I molar relation on both sides, with stable intercuspation between the upper and lower teeth, was reached. The upper right impacted canine was guided into the place of the missing upper right lateral incisor with healthy, sound periodontal tissue. The anterior and posterior crossbites, together with the lower midline shift, were eliminated. The periodontal tissues and the surrounding bone were found to be healthy (Figure 4).

**Figure 5:**
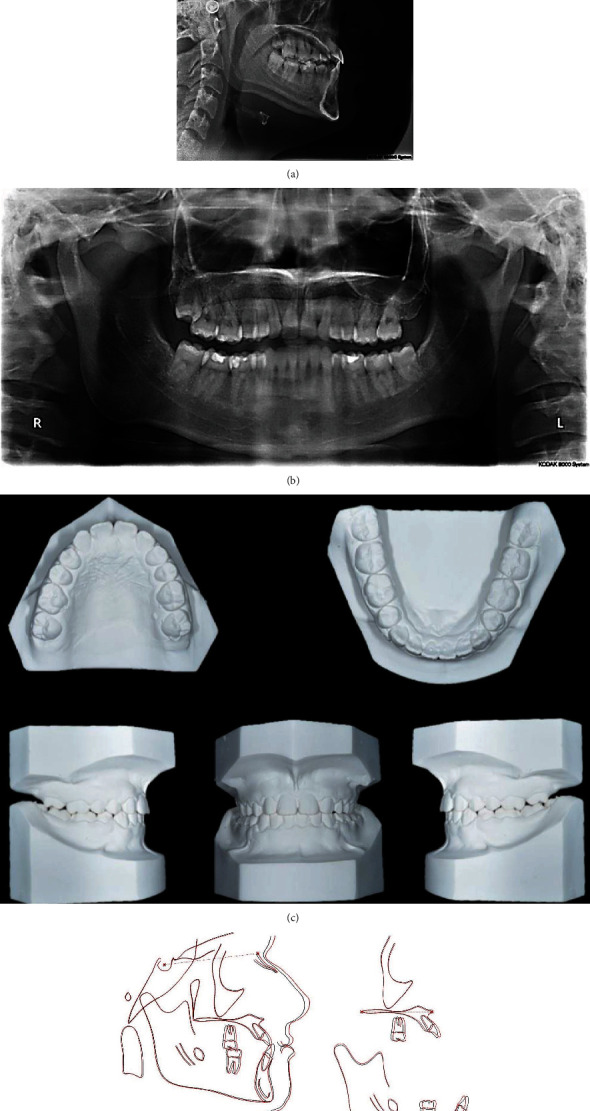
Posttreatment cephalometric radiograph, panoramic radiograph, study models, and superimposition: (a) cephalometric radiograph, (b) panoramic radiograph, (c) study models, and (d) cephalometric superimposition.

Surprisingly, the cephalometric analysis showed obvious skeletal changes in both the anteroposterior and vertical measurements. All the teeth showed normal bone levels with no signs of root resorption in the panoramic radiograph (Table 1) (Figures 5(a)–5(d)).

## 7. Discussion

Adult patients suffering from class III malocclusions may be treated either by orthognathic surgery or by orthodontic camouflage. The degree of severity of this malocclusion usually determines which treatment is pursued [15]. In this case, camouflage treatment successfully achieved the desired goals of the treatment. Considerable skeletal and soft tissue changes were observed after orthodontic treatment. These changes can be explained by the alveolar bone remodeling, which usually follows the orthodontic tooth movement. These changes were reported in numerous studies that indicated possible bone remodeling in adult patients after orthodontic treatment [16–19].

The impacted canine in this case may have been caused by the congenitally absent upper lateral incisor, as suggested by the guidance or genetic theory [20]. This could also explain the presence of the retained deciduous teeth; however, this could not explain why the left upper canine was not impacted, although the lateral was also missing on the other side. Furthermore, the unilateral posterior crossbite could have been originated from relative narrowing of the maxillary arch, which would eventually cause a cusp-to-cusp occlusion on the posterior teeth. This position is not usually stable for the mandible. The instability of this position usually guides the mandible to a lateral functional shift. After puberty, this functional shift usually becomes skeletal, which necessitates treatment by camouflage or orthognathic surgery [9].

Two treatment options were available for this case. The option that includes orthognathic surgery was rejected by the patient, and the severity of the case did not justify this option as camouflage treatment represents a less-invasive alternative with a relatively comparable outcome [21]. The substitution of the congenitally missing lateral incisor with the canines was also chosen in the treatment of this case. There were many reasons to prefer this option. The first reason is that from the biomechanical point of view, it would be faster and easier to allow for the eruption of the right upper canine into the space or upper right lateral as the crown of the impacted canine was already reaching the space of the upper lateral incisor. The second reason was to decrease the duration and cost of treatment. Additionally, if a bridge is utilized, it will require reduction of the neighboring teeth, which is regarded as a nonconservative solution. On the other hand, in areas with congenitally missing laterals, dental implants may require bone grafts due to a hypoplastic alveolar bone caused by the absence of the lateral incisor, which may require additional surgical phases and result in added costs to the patient [22].

The closed eruption technique was chosen to guide the upper right canine into its normal position. This technique was done because of the relatively high position of the canine. Additionally, it was found that the impacted canine usually shows better periodontal health when utilizing the closed eruption, compared to canines managed with the open method [23, 24].

## 8. Conclusion

In this case report, we found that combined skeletal problems in the anteroposterior and transverse dimensions, together with a congenital absence of teeth and impaction, could be efficiently managed by orthodontic camouflage. However, these results could not be achieved without utilizing reliable and evidence-based methods for diagnosis and treatment planning.

## Figures and Tables

**Table 1:** Cephalometric analysis.

	Measurement	Mean (±SD)	Initial	Final
Skeletal	SNA (°)	82^o^ (±3.3)	82.5	85.1
	SNB (°)	80^o^ (±3.1)	85.8	87.1
	ANB (°)	2^o^ (±1.7)	-3.4	-2
	Wits (mm)	*M* = −1.17 (±1.9)	-5.3	-0.8 mm
		*F* = −0.10 (±1.77)		
	Facial angle = NPg : FH (°)	87.8^o^ (±3.6)	94	91
	Angle of convexity NA-APg (°)	0^o^ (±5.1)	-8	-6
	MP (Go-Gn) : SN (°)	32^o^ (±3.5)	25	22
	MP (tangent lower border) : FH (°)	21.9^o^ (±3.2)	31	19
	Pg : NB (mm)	4(±2)	6 mm	2.2 mm
	*Y* axis (SGn : FH)	59.4^o^ (±3.8)	62	61
Dental	U1 to NA (°)	22^o^ (±6.1)	41	38
	U1 to NA (mm)	4 (±1.2)	7 mm	6 mm
	L1 to NB (°)	25^o^ (±4.5)	28.5	25
	L1 to NB (mm)	4 (±1.5)	6 mm	4 mm
	U1 to L1 (°)	131.7^o^ (±6.5)	113	109
	(Avg. Downs & Steiner)			
	L1: APg (mm)	1 (±2)	8 mm	4 mm
	IMPA (°)	90^o^ (85-95)	98	95
Soft tissue	Nasolabial angle (°)	90-110^o^	125	115
	Esthetic plane (E-line)	-4 mm	-8 mm	-5 mm
	Upper lip			
	Esthetic plane (E-line)	-2 mm	-2 mm	-3 mm
	Lower lip			

**Table 2:** Optimizing dental aesthetics when a maxillary canine is substituting for a lateral incisor and 1^st^ premolar is substituting for a canine.

(i) Localized vital bleaching or veneering
(ii) Extrusion of canine and intrusion of first premolar for correct anterior marginal gingiva
(iii) Reshaping the tip of a canine by grinding or composite build up plus reduction of labial enamel
(iv) Applying palatal root torque for correct crown positioning and to reduce buccal prominence of the canine root. This can be achieved by inverting the bracket if a minor (−7°) torque prescription canine bracket is being used
(v) Also labial root torque on the first premolars to mimic the canines
(vi) Reducing the width of the canine
(vii) Increasing the length of the buccal cusp of the first premolar by composite build-up or veneering

## Data Availability

No data were used to support this study, only the case which has been done in the Orthodontic Department, Dentistry College, King Abdulaziz University.

## References

[1] Rabie A.-B. M., Wong R. W., Min G. (2008). Treatment in borderline class III malocclusion: orthodontic camouflage (extraction) versus orthognathic surgery. *The open dentistry journal.*.

[2] de Lima E., Brum F., Mezomo M., Pasquali C. E., Farret M. (2017). Orthodontic treatment of class III malocclusion with lower extraction and anchorage with mini implants: case report. *Journal of the World Federation of Orthodontists.*.

[3] Chung K., Kim Y., Jeon H., Kim S., Nelson G. (2018). The biocreative strategy. Part 6: class III treatment. *Journal of clinical orthodontics: JCO.*.

[4] Tekale P. D., Vakil K. K., Sastri M. R. (2015). Correction of severe deep bite and gummy smile using mini-screw anchorage: a case report. *Journal of the World Federation of Orthodontists.*.

[5] Dhar S. (2019). Camouflage of skeletal class III malocclusion in an adult male using miniscrew anchorage from the external oblique ridge in conjunction with face mask wear. *Journal of Indian Orthodontic Society.*.

[6] Clemente R., Contardo L., Greco C., Di Lenarda R., Perinetti G. (2018). Class III treatment with skeletal and dental anchorage: a review of comparative effects. *BioMed Research International*.

[7] Haniyah F., Sunarto H., Tadjoedin F. M. (2018). Relationship between crossbite and periodontal status. *Journal of International Dental and Medical Research*.

[8] Krishna V., Sivakumar A., Indumathi S., Sam P. M., Padmapriya C. (2019). Treatment of 3-prong anterior crossbite and unilateral lingual posterior crossbite malocclusion in an adolescent boy. *Journal of Indian Orthodontic Society*.

[9] Gossman W., Palla A. (2019). *Orthodontics, Posterior Crossbite*.

[10] Tseng L. L., Chang C. H., Roberts W. E. (2016). Diagnosis and conservative treatment of skeletal class III malocclusion with anterior crossbite and asymmetric maxillary crowding. *American Journal of Orthodontics and Dentofacial Orthopedics*.

[11] Laurenziello M., Montaruli G., Gallo C. (2017). Determinants of maxillary canine impaction: retrospective clinical and radiographic study. *Journal of Clinical and Experimental Dentistry*.

[12] Afify A. R., Zawawi K. H. (2012). The prevalence of dental anomalies in the Western region of Saudi Arabia. *ISRN Dentistry*.

[13] Bassiouny D. S., Afify A. R., Baeshen H. A., Birkhed D., Zawawi K. H. (2016). Prevalence of maxillary lateral incisor agenesis and associated skeletal characteristics in an orthodontic patient population. *Acta Odontologica Scandinavica.*.

[14] Bertl M. H., Foltin A., Lettner S. (2018). Association between maxillary lateral incisors' root volume and palatally displaced canines: An instrumental variables approach to the guidance theory. *The Angle Orthodontist*.

[15] Proffit W. R., Phillips C., Douvartzidis N. (1992). A comparison of outcomes of orthodontic and surgical-orthodontic treatment of class II malocclusion in adults. *American Journal of Orthodontics and Dentofacial Orthopedics.*.

[16] Sharma J. N. (2010). Skeletal and soft tissue point a and B changes following orthodontic treatment of Nepalese class I bimaxillary protrusive patients. *The Angle Orthodontist.*.

[17] Ahn J.-G., Schneider B. J. (2000). Cephalometric appraisal of posttreatment vertical changes in adult orthodontic patients. *American Journal of Orthodontics and Dentofacial Orthopedics.*.

[18] Jiang C., Liu Y., Cheng Q. (2018). Chin remodeling in a patient with bimaxillary protrusion and open bite by using mini-implants for temporary anchorage. *American Journal of Orthodontics and Dentofacial Orthopedics.*.

[19] Hong S. Y., Shin J. W., Hong C. (2019). Alveolar bone remodeling during maxillary incisor intrusion and retraction. *Progress in Orthodontics*.

[20] Peter E. (2016). Genetic causes vs guidance theory for palatal displacement of canines. *American Journal of Orthodontics and Dentofacial Orthopedics.*.

[21] Troy B. A., Shanker S., Fields H. W., Vig K., Johnston W. (2009). Comparison of incisor inclination in patients with Class III malocclusion treated with orthognathic surgery or orthodontic camouflage. *American Journal of Orthodontics and Dentofacial Orthopedics*.

[22] Muhamad A.-H., Nezar W., Azzaldeen A. (2016). Managing congenitally missing lateral incisors with single tooth implants. *Dental, Oral and Craniofacial Research*.

[23] Kumar P., Datana S., Kotwal A., Saxena V. (2014). Guided tooth eruption: comparison of open and closed eruption techniques in labially impacted maxillary canines. *Journal of Dental Research and Review*.

[24] Abu-Hussein M. (2016). Congenitally missing lateral incisors; orthodontic, restorative, and implant approaches. *International Journal of Dentistry*.

